# Skeletal muscle wasting and renewal: a pivotal role of myokine IL-6

**DOI:** 10.1186/s40064-016-2197-2

**Published:** 2016-05-13

**Authors:** José E. Belizário, Cibely C. Fontes-Oliveira, Janaina Padua Borges, Janete Akemi Kashiabara, Edouard Vannier

**Affiliations:** Department of Pharmacology, Institute of Biomedical Sciences, University of São Paulo, Avenida Lineu Prestes, 1524, São Paulo, SP 05508-900 Brazil; Unit of Muscle Biology, Lund University, BMC B12, 221 84 Lund, Sweden; Division of Geographic Medicine and Infectious Disease, Tufts Medical Center, Boston, MA 02111 USA

**Keywords:** IL-6, Skeletal muscle, Satellite cells, Exercise, Cancer cachexia, IL-6/IL-6R/gp130 signaling pathway, Jak/STAT signaling, Anti-IL-6 receptor monoclonal antibodies

## Abstract

Adult skeletal tissue is composed of heterogeneous population of cells that constantly self-renew by means of a controlled process of activation and proliferation of tissue-resident stem cells named satellite cells. Many growth factors, cytokines and myokines produced by skeletal muscle cells play critical roles in local regulation of the inflammatory process and skeletal muscle regeneration during different pathological conditions. IL-6 is a pleiotropic cytokine released in large amount during infection, autoimmunity and cancer. Low levels of IL-6 can promote activation of satellite cells and myotube regeneration while chronically elevated production promote skeletal muscle wasting. These distinct effects may be explained by a crosstalk of the IL-6/IL-6 receptor and gp130 trans-signaling pathway that oppose to regenerative and anti-inflammatory of the classical IL-6 receptor signaling pathway. Here we discuss on potential therapeutic strategies using monoclonal antibodies to IL-6R for the treatment of skeletal muscle wasting and cachexia. We also highlight on the IL-6/JAK/STAT and FGF/p38αβ MAPK signaling pathways in satellite cell activation and the use of protein kinase inhibitors for tailoring and optimizing satellite cell proliferation during the skeletal muscle renewal. Future investigations on the roles of the IL-6 classical and trans-signaling pathways in both immune and non-immune cells in skeletal muscle tissue will provide new basis for therapeutic approaches to reverse atrophy and degeneration of skeletal muscles in cancer and inflammatory diseases.

## Background

Interleukin (IL)-6 is one of hundreds cytokines, chemokines and growth factors that function as mediators of the innate and adaptive immune responses (Pal et al. 2014; Hunter and Jones 2015). In addition, IL-6 exerts endocrine and metabolic functions on various organs including liver, fat, gut, pancreas and skeletal muscle. Recent studies have revealed the pivotal role of IL-6 during exercise-induced skeletal muscle injury and repair (Pedersen 2000; Benatti and Pedersen 2014). Nonetheless, under some pathological conditions, IL-6 leads to muscular atrophy (Tsujinaka et al. 1996; Baltgalvis et al. 2008). Acting at skeletal muscle tissue IL-6 may promote the proliferation of satellite cells and their incorporation as new myonuclei into existing fiber syncytia (Cantini et al. 1995). Although the direct link between these phenomena has not fully established in vivo, exercise and anti-IL-6 receptor based approaches have now been considered as valuable therapeutic strategies to combat muscle wasting associated with chronic inflammatory conditions (Ardies 2002; Narsale and Carson 2014). Here we will review emerging concepts and mechanisms that underlie the complex biology of IL-6 in regard to its roles in cancer cachexia and skeletal muscle renewal. Finally we will outline the benefits and side effects that therapy with monoclonal antibody to IL-6 and IL-6R may bring to patients undergoing skeletal muscle wasting.

## Satellite cells and skeletal muscle renewal

Skeletal muscle is a very dynamic and heterogeneous tissue of adult human body (Bassel-Duby and Olson 2006; Bentzinger et al. 2012). Striated muscle represents approximately 40 % of total body weight and plays diverse roles in the whole-body metabolism (Bassel-Duby and Olson 2006; Muscaritoli et al. 2010). This tissue is considered an endocrine organ as it produces and secretes growth factors, cytokines and peptides, referred to as myokines (Pedersen and Febbraio 2012). Many myokines have been described by their ability to exert positive and negative effects on the skeletal muscle self-renewal (Muñoz-Cánoves et al. 2013; Pal et al. 2014).

Adult skeletal muscle fibers consist of multinucleated differentiated myocytes that adapt to physiological demands imposed by body growth, physical training and trauma. Damaged skeletal muscle are repaired and replaced (Yablonka-Reuveni et al. 2008). The ability to repair and maintain skeletal muscle is attributed to satellite cells, the muscle-resident myogenic stem cells which are formed during embryonic development and sit at satellite position outside the myofiber under the basal lamina and account for approximately 5 % of the cell mass in adult skeletal muscle (Bentzinger et al. 2012; Yin et al. 2013; Chang and Rudnicki 2014).

**Fig. 1:**
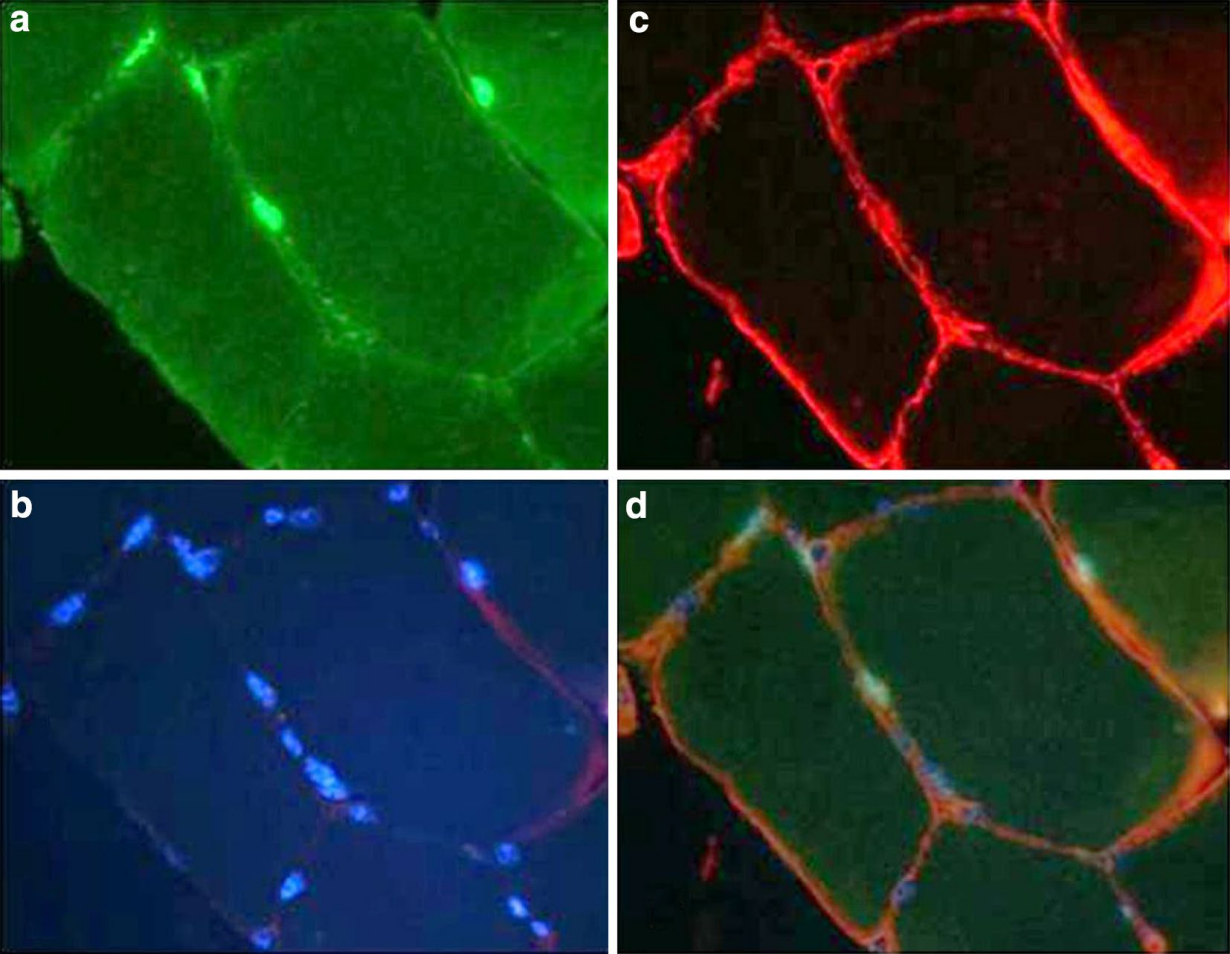
Immunohistochemical identification of satellite cells and myonuclei in human *vastus lateralis muscle*. Satellite cells are distinguished using co-staining for Pax7, laminin and DNA. Panels are images of a representative cross-section of skeletal muscle tissue stained for: Pax7 (**a**), myonuclei with Hoescht 33342 (**b**), laminin (**c**) and the merge image (**d**). Adapted from Brooks et al. 2010

Satellite cell populations are comprised of satellite stem cells and satellite myogenic cells; each population is characterized by a set of distinct markers (Yin et al. 2013; Motorashi and Assakura 2014). In adult skeletal muscle, most of satellite cell populations express the paired domain transcription factors Pax7 and Pax3, and myogenic regulatory factors MyoD and MyF5 in the nucleus. They also express at the membrane the proteins: caveolin-1, cell surface attachment receptor intergin α7, transmembrane heparan sulfate proteoglycans: syndecan-3 and -4, cluster of differentiation protein 34 (CD34) and the calcitonin receptor, which can vary at some point during the development across multiple species (Seale and Rudnicki 2000; Seale et al. 2000; Yin et al. 2013). Figure 1 shows an immunohistochemical assay for identification of satellite cells in human *vastus lateralis muscle*. The satellite cells are identified as Pax7 positive cells with nuclei stained blue with Hoescht 33342. They are located within the basal lamina as revealed by laminin expression. Further details are described in Brooks et al. (2010). These and other biomarkers contribute intrinsically to different properties of satellite cells depending on their origin (Brooks et al. 2010; Yin et al. 2013). The basic helix-loop-helix family of muscle regulatory transcription factors (MRF): Myf5, Myf6, Myod1 (MyoD) and myogenin play key roles in satellite cell commitment for myotube formation (Kollias and McDermott 2008; Chang and Rudnicki 2014). MyoD and Myf5 are activated by Pax7 which is mainly expressed in quiescent satellite cells and is co-expressed with MyoD during myoblast proliferation, but declines during differentiation due to activation of myogenin (Elia et al. 2007).

In adulthood, satellite stem cells remain in quiescent state and are activated in response to specific signals that promote their exit from the basal lamina. Satellite cell activation causes their symetric and asymmetric cell division, which produce stem and committed progenitors (Seale and Rudnicki 2000; Shi and Garry 2006; Motorashi and Assakura 2014). Once properly stimulated, these cells proliferate, differentiate and fuse into new or existing myotubes, thereby facilitating skeletal muscle repair or overgrowth (Yablonka-Reuveni et al. 2008). Satellite cells are located at specific niches of skeletal muscle tissue where specific chemical microenvironment exists to maintain their quiescence and undifferentiated state (Boonen and Post 2008; Yin et al. 2013). Changes in this microenvironment, in response to physiological and/or pathological stimuli, allow escape from the quiescent state, and initiation of activation and differentiation processes into myotubes (Gopinath and Rando 2008). Disruption of proliferative capacity and reduced quiescent cells populations are linked to aging and muscle dystrophies (Carlson and Conboy 2007; Brien et al. 2013). Loss of regenerative capacity in skeletal muscles is caused by excessive cycles of degeneration, regeneration and increased interstitial fibrosis. One study elegantly demonstrated that satellite cells are essential for skeletal muscle regeneration using mdx mouse model as surrogate of Duchenne muscular dystrophy, which is caused by a point-mutation in dystrophin gene (Luz et al. 2002).

The contribution of endogenous satellite cells to muscle regeneration has clearly been demonstrated using the Cre-lox system in mice as this approach allows sophisticated temporal control of *pax3, pax7, myf5 and myoD* gene expression and/or deletion (Relaix and Zammit 2012; Yin et al. 2013). *Myod1*^−/−^ mutant mice display markedly reduced musculature due impaired skeletal muscle cell differentiation (Megeney et al. 1996). In contract, Myf5^−/−^ mutant mice display myofiber hypertrophy and impairment in the myoblast proliferation (Gayraud-Morel et al. 2007). The inactivation of both *Myod1 and Myf5* genes in double knockout transgenic mice results in complete loss of formation of skeletal muscle (Gayraud-Morel et al. 2007; Bryson-Richardson and Currie 2008). Recently, experiments using a parabiotic pair of young and old mice have suggested that factors present in the circulation of young mice are capable of inducing the rejuvenating activity and muscle repair in aged mice (Blau et al. 2015). GDF11, a transforming growth factor beta (TGF-β) superfamily member, is a putative systemic rejuvenation factor (Blau et al. 2015). These studies have been a matter of intense debate in the literature (Brack and Muñoz-Cánoves 2016).

Several family of growth factors including Shh (sonic hedgehog embryonic factor), Noggin, fibroblast growth factor family member (FGFs) and transforming growth factor-beta family members (TGF-β) are required for proliferation and differentiation of skeletal muscle satellite cells into myotubes (Parker et al. 2003; Zammit et al. 2006). Shh binds to the receptor PTCH1 (Patched1) and activates protein Smo (Smoothened). Smo induces the activation of the transcription factor Gli, which translocate to the nucleus, increasing the transcription of MRFs, which control the activation of muscle differentiation-specific genes, including Myf5 (Halevy et al. 1996; Riobo et al. 2006; Bryson-Richardson and, Straface et al. 2009). Even though Shh signaling pathway does not appear to be activated in postnatal life, it was demonstrated that this pathway can be reactivated during skeletal muscle ischemia and infarction (Kusano et al. 2005). FGFs have multiple isoforms which participate in the proliferation of activated satellite cells via the Ras–Raf–MEK–ERK signaling pathway (Shi and Garry 2006; Carlson and Conboy 2007). The activation of both p38α and p38β mitogen-activated kinase pathway is required to progression of embryonic progenitor cells into the myogenic program (Shi and Garry 2006; Carlson and Conboy 2007).

During skeletal muscle inflammatory process, immune cells, in particular, neutrophils, eosinophils and macrophages produce many growth factors; cytokines, lipids mediators, as well as damage-associated molecular patterns released by death cells, have a great impact on satellite cell behavior and the skeletal muscle repair process (Kharraz et al. 2013). Interleukin (IL)-6 is one of important mediator that play a pivotal role in the regenerative and anti-inflammatory processes (Cantini et al. 1995).

## Interleukin-6

IL-6 is secreted as glycoprotein of 21–28 kDa by both lymphoid and non-lymphoid cells and participate in many leukocyte functions, hematopoiesis and acute phase reactions (Pal et al. 2014; Hunter and Jones 2015). IL-6 belongs to the granulocyte colony-stimulating factor-like protein family of cytokines (Pal et al. 2014). The superfamily of IL-6 includes IL-6 itself, IL-11, IL-27, IL-31, leukemia inhibitory factor (LIF), ciliary neurotrophic factor (CNTF), oncostatin M (OSM), cardiotropin-1 (CT-1), neuropoietin (NPN) and cardiotropin-like cytokine (CLC) (Lahiri et al. 2001; Skiniotis et al. 2008; Chalaris et al. 2011). These cytokines and growth factors bind to specific receptors and activate a series of target genes involved in cell proliferation, cell differentiation, apoptosis and pro- and anti-inflammatory biochemical processes. The glycoprotein 130 (gp130 or CD130), a membrane-bound co-receptor is the major member of the family of ‘‘tall’’ cytokine receptors which homodimerize or heterodimerize with several receptors in the family performing a central role in the activation of intracellular signaling pathways (Heinrich et al. 2003; Skiniotis et al. 2008; Pal et al. 2014; Wolf et al. 2014). The structural model and the architecture of the homo- and heterodimer complexes and multiple contact sites within receptors and gp130 are presented and discussed in detail elsewhere (Skiniotis et al. 2008).

**Fig. 2:**
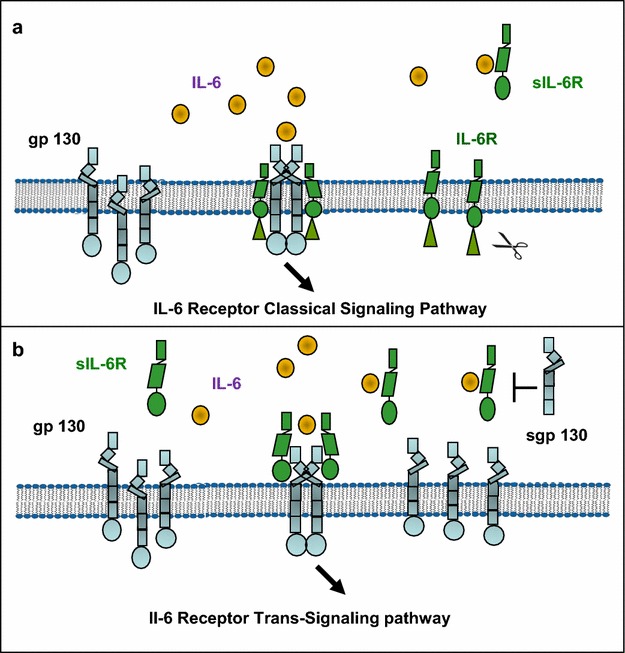
IL-6 classical and trans-signaling pathways. **a** IL-6 binds to cells that express both the membrane bound IL-6R gp130 and trigger the activation of the JAK/STAT signaling pathway. This type of signaling is called classical signaling. **b** In cells that express only gp130 but not IL-6R, IL-6 binds to soluble IL-6R (sIL-6R) and the complex in turn bind to gp130 to trigger the activation of intracellular signaling. This type of signaling is called trans-signaling. The sIL-6R is released by proteolytic cleavage of the IL-6R membrane bound precursor by the metalloproteases ADAM10 and 17. A natural form of gp130 is able to bind to the sIL-6R bound to IL-6 with comparable affinity and mediates inhibition of IL-6 trans-signaling

IL-6 exerts its biological activities through two molecules: IL-6 receptor alpha (IL-6Rα or CD126) and gp130 (Fig. 2). The classical IL-6Rα activation is initiated upon binding of IL-6 to the two membrane-bound p80 receptors that is followed by recruitment and homodimerization of the gp130. The hexameric complex cause the phosphorylation of gp130 intracellular tyrosine residues and transcription factors STAT (signal transducer and activators of transcription) by Janus kinases JAK1, JAK2, TYK2 (Heinrich et al. 2003; Matsushita et al. 2005; Hunter and Jones 2015). STATs translocate into nucleus and bind to DNA promoters of target genes. The IL-6R/gp130 receptor complex also leads to activation and the Ras-Raf-ERK (extracellular signal regulated kinase)/MAPK (mitogen-activated protein kinase) pathway and the PI3K (phosphoinositide 3-kinase) and the serine/threonine protein kinase B/Akt pathway (Pedersen et al. 2001; Kallen 2002). The net biological activity of IL-6 depends on the formation of a particular functional receptor complexes and a variety of downstream biochemical regulatory events. Three families of regulators of JAK/STAT signaling are known: the SOCS (suppressor of cytokine signaling) family, the PIAS (protein inhibitor of activated STAT) family and the SHP2-containing phosphatase family, which are proteins that interact with distinct downstream effectors (Heinrich et al. 2003; Matsushita et al. 2005). SOCS3 is a classical feedback inhibitor of STAT3 activation involved in signaling pathways that determine the synthesis of a wide range of target genes in liver and skeletal muscle (Skiniotis et al. 2008).

The expression of IL-6R gene is restricted to some cell types; however IL-6 can act on a large range of cells and tissues (Mullberg et al. 2000; Kallen 2002). The ubiquitous activity of IL-6 is explained by the unrestricted expression of gp130 on many cell types that do not express membrane-bound IL-6R (Mullberg et al. 2000; Kallen 2002; Scheller et al. 2006; Fasnacht and Muller 2008; Schellera et al. 2011). According to data of many studies, IL-6 interacts with soluble IL-6R and this complex associated with the membrane-bound gp130 triggering JAK/STAT pathway (Kallen 2002; Scheller et al. 2006; Schellera et al. 2011). This mechanism of transmission is called trans-signaling (Fig. 1). IL-6R molecules are shedding of membrane bound IL-6Ra after cleavage by metallopeptidases ADAM-10 and -17. In cells that weakly express or do not express IL-6R, such as hematopoietic stem cells, neural cells and smooth muscles, trans-signaling is critical for IL-6 activity (Mullberg et al. 2000; Schellera et al. 2011). Although soluble receptors typically inhibit ligand activity by functioning as decoy moieties, sIL-6R itself promotes IL-6 activity. For example, sIL-6R inhibits cardiomyocyte apoptosis in a mouse model of myocardial infarction (Matsushita et al. 2005).

**Fig. 3:**
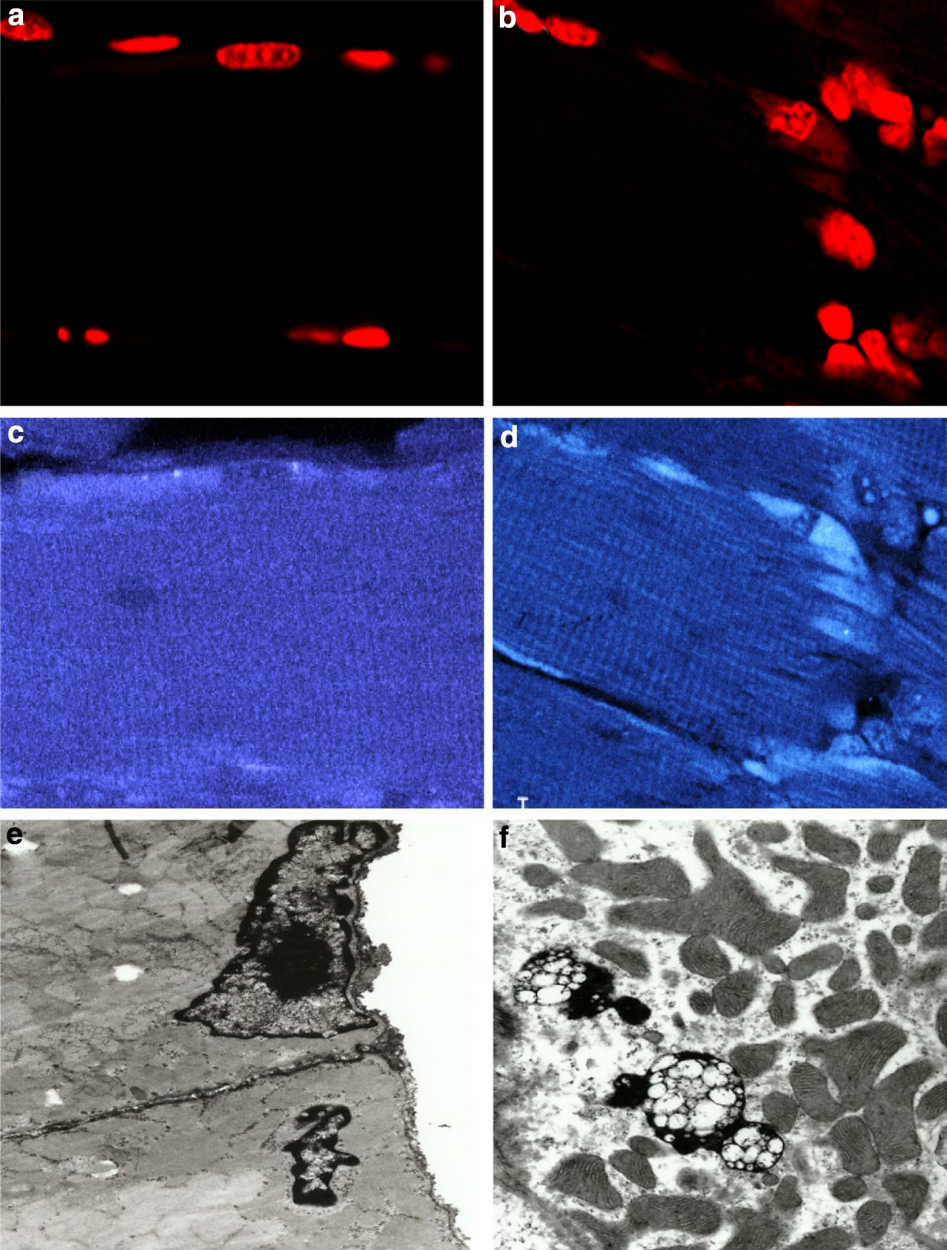
Skeletal muscle morphological alterations in cancer cachexia revealed by confocal (**a**–**d**) and transmitted electron (**e**, **f**) microscopic examination of a cross section of soleus muscle from mouse bearing B16 melanoma and severe cachexia as compared to normal soleus obtained from control C57BL/6 mouse. Myocyte apoptotic cell death is characterized by DNA fragmentation and deposition of chromatin masses around nuclear membrane. In **a**, a representative image illustrating the normal flattened nuclei located in parallel with skeletal muscle fibers in the periphery of normal myotube. In **b**, the image shows irregular nucleus with visible clumping of nuclear chromatin confirming cell death by apoptosis. In **c**, the micrograph shows the myosin banding displaying regular cross-striations and intense fluorescence. In **d**, illustrates myosin appearance in cachectic skeletal muscle fiber. The weak fluorescence intensity is reflecting breakdown of myofibril and tissue architecture. Nuclei were stained with propidium iodide and myosin pattern was revealed with monoclonal antibody to fast myosin skeletal heavy chain (*lower panel*). In **e**, the electron micrograph shows a nucleus with normal morphology (*upper side*) and a typical apoptotic nucleus with condensed chromatin fragments at its periphery (*lower side*). In **f**, the micrograph shows a portion of sarcolemma surrounded by mitochondria and vacuoles containing apoptotic bodies (autophagosome). The ubiquitin–proteasome and autophagy-lysosome pathways are the two major routes for protein and organelle degradation along skeletal muscle wasting

Regulation of IL-6 biological activities goes beyond soluble IL-6Rα. Complex IL-6/sIL-6R can engage membrane-bound gp130 but also its soluble form (sgp130) that in turn acts as a decoy receptor (Scheller et al. 2006; Schellera et al. 2011). The membrane IL-6R and gp130 levels can dictate whether sIL-6R acts as agonist or antagonist. For cells with comparable IL-6R and gp130 levels, IL-6 activity is not inhibited by sIL-6R. In cells that IL-6R is highly expressed than gp130, the addition of sIL-6R, inhibit the response to IL-6 because of the formation of an inactive complex containing only one molecule of gp130 (Scheller et al. 2006).

## Exercise increases IL-6 synthesis by skeletal muscle cells

Exercise is a complex process involving the synchronized and integrated activation of multiple tissues and organs, in particular, musculature, at the cellular and systemic level (Hawley et al. 2014). Following progressive exercise training, skeletal muscle mass is increased due to net gain in protein synthesis (Russell 2010). Regular physical exercise has a protective effect against various diseases such as cardiovascular diseases and obesity. Moreover, it increases life span and retards appearance of illness in elderly (Pedersen 2000, 2011; Ardies 2002). Regular exercise and physical activity is associated with specific production of cytokines and myokines, including IL-1β (Cannon et al. 1986), IL-6, TNF-α, TNF receptors, IL-10, IL-8, IL-1 receptor antagonist and acute phase proteins (Pedersen 2000; Febbraio and Pedersen 2002) by the skeletal muscle to maintain the metabolic homeostasis of lipids and proteins. Many studies have reported on the biological effects exerted by myokines to increase the energy supply including glucose uptake via translocation of GLUT4 (Weigert et al. 2006); as well as to increase the synthesis of glycogen (van Hall et al. 2003) and hepatic production of glucose and its absorption by the intestine epithelial cells (Pedersen et al. 2001; Egan and Zierath 2013).

IL-6 mediates many aspects of the exercise-induced acute-phase response, including the up regulation of antioxidant defenses as response to oxidative stress (Sacheck et al. 2008). After strenuous physical exercise, IL-6 is synthetized by actively contracting muscle fibers and increase up to 100-fold in relation to other pro-inflammatory cytokines such as TNF-α and IL-1β (Pedersen 2000; Pedersen et al. 2004; Benatti and Pedersen 2014). It has been proposed that IL-6 and other myokines produced in response to strenuous and prolonged exercise lead to increased proliferation of satellite cells and thereby regeneration of damaged myofibers (Cantini et al. 1995; Steensberg et al. 2001). Serrano et al. demonstrated that muscle hypertrophy under the action of IL-6 requires STAT3 activation (Serrano et al. 2008). This study showed a clear evidence for the contribution of satellite cells using mice lacking *IL*-*6* gene in skeletal muscle (Serrano et al. 2008). STAT3 play a critical role during development and its deletion lead to early embryonic lethality (Takeda et al. 1997). STAT3 has been examined a possible target for skeletal muscle repair since it is critical for MyoD1 mediated transcription of myogenesis genes (Tierney et al. 2014). It was observed that only one cyclic or transient administration of STAT3 inhibitors in aged, injured or dystrophic skeletal muscles can promote myofiber repair. One explanation for the positive results is that partial STAT3 inhibition, by augmenting repeated rounds of satellite cell expansion, may prevent the exhaustion of reservoir of the muscle satellite cells (Tierney et al. 2014). This mechanism need to be explored in response to long term training. It is interesting that the skeletal muscle tissue of mice genetically deficient of myostatin gene (Mstn^−/−^) retains the same metabolic plasticity and adapted normally to endurance training (Savage and McPherron 2010). This is consistent with the results of recent study showing that muscle satellite cells do not play any role in skeletal muscle hypertrophy induced by inhibition of the myostatin/activin signaling pathway or deletion of *Acvr2b* gene which encodes a high-affinity receptor for myostatin and activin A (Lee et al. 2012).

## IL-6 induces muscle wasting and cancer cachexia

The development of neoplastic disorders is linked to biochemical changes in the body, which include reduction of the nutrient intake of nutrients and stimulation of catabolic pathways, and ultimately to the onset of cachexia (Baracos and Mackenzie 2006; Fearon et al. 2011). Cachexia is defined as a multi-factorial syndrome (Evans et al. 2008; Argiles et al. 2010). This syndrome is present in patients with cancer and various chronic diseases among them, AIDS, diabetes, kidney failure and heart failure (Rubin 2003; Tan and Fearon 2008; Muscaritoli et al. 2010). Cachexia accounting for 20–50 % of the deaths of these patients (Tan et al. 2008; Muscaritoli et al. 2010; Fearon et al. 2011). Cachexia reduces the patient’s response to chemotherapy and radiotherapy (Laine et al. 2013), and the weight loss is inversely proportional to survival time and directly related to worse prognosis and poor quality of life of patients (Fearon 2008; Tan and Fearon 2008; Fearon et al. 2011; Laine et al. 2013; Argiles et al. 2014a).

Skeletal muscle morphological alterations in cancer cachexia (see Fig. 3 as example) have been attributed to many factors and mechanisms depend on cancer cell type, animal model and the experimental protocol. Cachexia promotes a drastic reduction of body weight due to inhibition of protein synthesis and increased degradation, especially, of skeletal muscle proteins (Inui 1999; Ventrucci et al. 2002; Schiaffino et al. 2013). Several hormones, growth factors, pro-inflammatory cytokines and humoral factors have been described as potential mediators of muscle mass loss (Tisdale 2004; Fearon et al. 2012; Argiles et al. 2014b). There is strong evidence that cachexia in patients with cancer involves an inflammatory process with an increased release of pro-inflammatory and anti-inflammatory cytokines (Argiles et al. 2005; Seelaender et al. 2012). The presence of these cytokines leads to anorexia, weight loss, changes in the metabolism of lipids and proteins, increased concentrations of catabolic hormones, reduction of anabolic hormones and metabolic impairment (Rubin 2003; Argiles et al. 2005; Seelaender et al. 2012). In fact, several studies have shown that TNF-α, IL-1, IL-6 and interferon gamma (IFN-γ), released by both tumor cells and the host in response to the tumor may lead to activation of different pathways of intracellular protein degradation (Tisdale 2004, 2009). Cachetin/TNF-α was the first cytokine identified that was able to reproduce most of the symptoms of cachexia in mice including changes in protein turnover and increased protein degradation (Oliff et al. 1987; Fong et al. 1989). IL-1α and β also contribute to protein degradation in rats and their administration also promoted weight loss and anorexia (Belizario et al. 1991; Shibata et al. 2000). It is interesting that the presence of IL-1β gene allele contributed to the occurrence of cachexia associated with gastric cancer in the Chinese population (Zhang et al. 2007).

IL-6 is capable of induce cachexia altering the metabolism of lipids and proteins as well as impairing myogenic differentiation in certain types of cancer (Jablons et al. 1989; Holmer et al. 2014; Narsale and Carson 2014; Pelosi et al. 2014). Studies undertaken with IL-6 transgenic mice and colon-26 tumor-bearing mice that display elevated plasma IL-6 levels have demonstrated about 25 % decrease in gastrocnemius muscle weight (Zhou et al. 2003). In patients with cancer cachexia, high circulating levels of IL-6 seems to act as mediator of skeletal muscle proteolysis (Tsujinaka et al. 1995). In fact, the overexpression of IL-6 in transgenic mice caused muscular atrophy and increased levels of cathepsin in skeletal muscle, indicating that IL-6 is involved in the regulation of muscle protein degradation (Tsujinaka et al. 1996). Haddad et al. (2005) showed that infusion of IL-6 causes muscle atrophy by a mechanism involving the reduction in phosphorylation of ribosomal protein kinase S6K1 and increased transcription of SOCS-3. Kwak et al. (2004) showed that in undifferentiated C2C12 myoblasts the treatment with IL-6 stimulates protein ubiquitination mainly by increasing the activity of ubiquitin ligase E3 α-II, suggesting that this cytokine contributes to the development of the process of cachexia. Recently, another study showed that IL-6 induces protein loss activating the JAK/STAT signaling pathway (Baltgalvis et al. 2009). Nonetheless, some authors consider that IL-6 act together with other cytokines thus has only a permissive role in the development of skeletal muscle proteolysis.

The TGFβ-superfamily members, including TGF-β1, GDF-8 (myostatin), GDF-11, GDF-15, activin A and B, nodal, BMP-2 and BMP-7 are well known by their role in the induction of muscle atrophy (Kollias and McDermott 2008; Glass 2010a, b). Myostatin (Mstn) inhibits differentiation of myoblasts via the Akt/mTOR pathway which promote inhibition of MyoD and myogenin synthesis (Glass 2010a, b). In several farm animals, mutation or deletion of *Mstn* gene increased 2–3 times skeletal muscle mass as compared to wild-type animals, thus highlighting its role in regulating hypertrophy and hyperplasia programs in the skeletal musculature (McPherron et al. 1997). Myostatin, upon binding to type II activin receptors (ACVR2 and ACVR2B), form a complex with a type I receptor, either activin receptor-like kinase 4 (ALK4) or ALK5, to stimulate the phosphorylation of the Smad2 and Smad3 transcription factors (Glass 2010a, b). ACVR2B is a high affinity activin type 2 receptor that mediates the intracellular signaling induced by myostatin, activin and GDF11 in skeletal tissue, whose inhibition attenuate cancer cachexia (Zhou et al. 2010). The blockade of ACVR2B through a direct neutralizing antibody has been approved for the treatment of multiple conditions associated with muscle wasting (Lach-Trifilieff et al. 2014). Insulin-like growth factor (IGF1) can counteract myostatin’s effects by rescuing of the PI3K/Akt pathway. It is interesting that ACVR2B inhibition also results in restoration of PI3K/Akt signaling (Lach-Trifilieff et al. 2014). Recently myostatin was identified in the secretome of C26 colon cancer cells and experimentally confirmed as novel tumoral factor that induces cancer cachexia via binding to ACVR2B (Lokireddy et al. 2012).

Many intracellular protein degradation systems are activated during skeletal muscle atrophy (Goldberg 2003). The ATP-ubiquitin–proteasome pathway play a principal role (Goldberg 2003; Lecker et al. 2006; Pickart and Cohen 2004). In this way, proteins are first marked for degradation by a covalent addition of an ubiquitin chain (known as multi-ubiquitination). This lead to a cascade of reactions that requires enzyme E1 (Ub activating), E2 enzymes (conjugated) and E3 (ligases) (Ciechanover 2003; Lecker et al. 2006). Several studies have shown that specific groups of genes involved in ubiquitin–proteasome system are induced during the loss of skeletal muscle in several models of cachexia and muscle atrophy (Lecker et al. 2004; Kandarian and Jackman 2006). The two transcription factors of FoxO family: FoxO1 and FoxO3 have been described as the main initiator of the transcription of genes involved in protein degradation during muscle atrophy (Sandri et al. 2004). FoxO1 and 3 participate in the activation of transcription of two E3 ligase genes: MuRF1 and MAFbx, which are selectively upregulated in skeletal and heart muscles (Bodine et al. 2001; Gomes et al. 2001). Myostatin induced skeletal muscle wasting through up-regulation of FoxO1 and MuRF1 (Lokireddy et al. 2012). MuRF1 binds to a large sarcomeric protein titin and myosin heavy chain (MyHC), thereby promoting their degradation via the proteasomes (Centner et al. 2001; Cohen et al. 2009; Lokireddy et al. 2012). However, ablation of MuRF1 or MAFbx only partially protects muscle loss during denervation (Bodine et al. 2001) and following glucocorticoid treatment (Baehr et al. 2011) in mice indicating that other ubiquitin ligases are also involved in skeletal protein degradation.

The degradation of membrane receptors and long-lived proteins during muscle atrophy is mediated by the lysosomal pathway in which the member of the cysteine family of aspartyl proteases cathepsins B, H, L and D, play predominant roles (Baracos et al. 1995; Costelli et al. 2005; Mammucari and Milan 2007; Attaix and Bechet 2007). Autophagy is a catabolic process that target damaged and no longer functional cellular organelles for lysosomal degradation. Autophagy play essential role in various myopathies and in response to reduction of the PI3K-Akt signaling pathway induced by IGF-1 and insulin (Bechet et al. 2005; Sandri 2012). Mammucari et al. (2007) and Zhao et al. (2007) demonstrated that many genes involved in autophagy such as Atg (Beclin 1), LC3 and Gabarapl1 are transcriptionally regulated by FoxO3 (Sandri 2013). The majority of these genes are induced along the processes of atrophy and muscle wasting after fasting, renal failure, diabetes, cancer and denervation in different animal models (Lecker et al. 2004; Zhao et al. 2007; Sandri 2013; Judge et al. 2014).

The myocyte cell death by apoptosis (Fig. 3), which is mediated by caspases, also contributes to muscle wasting (Sandri 2002; Argiles et al. 2005). The members of this family of cysteine proteases are activated by the extrinsic and intrinsic pathways of apoptosis (Duprez et al. 2009; Pop and Salvesen 2009). The inflammatory caspase-1, -4, -5, -11 and -12 are activated following inflammasome formation in response to microbe infection, cell stress and injury (Martinon and Tschopp 2007; Sangiuliano et al. 2014). The activity of caspase-1, -3, -8 and -9 is increased several folds in the skeletal muscle tissue of mice bearing MAC16, a cachexia-inducer tumor as compared to non-cachexia-inducer MAC13 (Belizário et al. 2001). Activation of caspase-3 is required for the cleavage of myofibrillar actin and possibly other myofibrillar and cytoplasmic proteins via the ubiquitin–proteasome system (Du et al. 2004; Wang and Mitch 2010; Silva et al. 2015). Caspase-3 also cleaves specifically the AAA-ATPase subunits of the 19S regulatory chamber of the proteasome (Wang and Mitch 2010; Silva et al. 2015). Biochemical studies have supported the hypothesis that the degradation and release of 19S unit from the 26S large unit promote the entry of non-ubiquitinated substrates into 20S catalytic chamber for their rapid degradation (Takeuchi et al. 2007; Belizario et al. 2008).

**Fig. 4:**
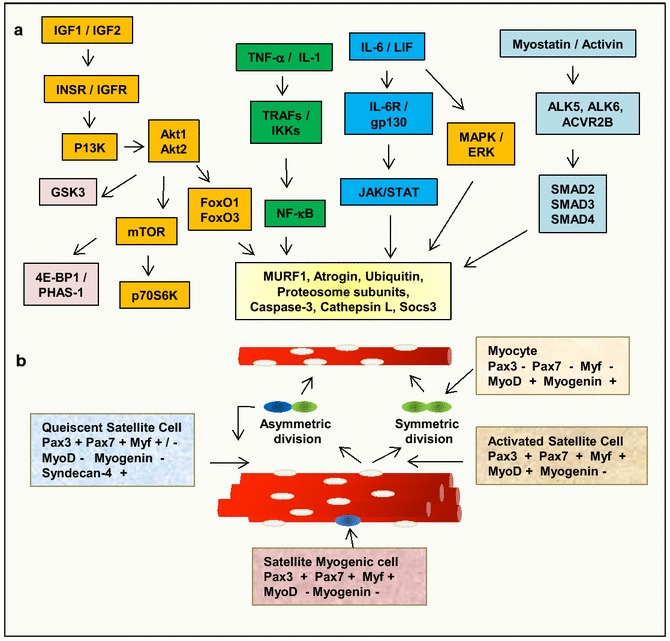
Mediators and signaling pathways involved in the control of myotube degradation and repairing via satellite cell activation. **a** Schematic representation of muscle growth and muscle wasting pathways generated in the studies of gene expression patterns in skeletal muscle from cancer cachexia mice models. Genes with growth-promoting activity in skeletal muscle are shown in *orange* and genes involved muscle wasting in *green* and genes involved in satellite cell activation in *blue*. This set of genes is significantly up-regulated during food deprivation, diabetes, uremia, and cancer cachexia and target genes under the control of FoxO transcription factors in skeletal muscle wasting. **b** Quiescent satellite cells (*white*) activated by myokines and injury initiate symmetric and asymmetric divisions to produce activated satellite cells (*green*) and self-renewing satellite cell and myogenic stem cells (*blue*). After many rounds of division their progeny differentiate into myocytes and then myotubes. A different set of the transcription factors and membrane protein are expressed along each differentiation state of satellite cells. Some specific biomarkers are indicated in the panels inside the figure. Adapted from Bonetto et al. (2014) and Yin et al. (2013). IGF insulin-like growth factor; INSR, insulin receptor substrate 1; IGFR, insulin-like growth factor receptor; PI3K, phosphoinositide 3-kinase; GSK3, Glycogen synthase kinase 3; 4E-BP1, Eukaryotic translation initiation factor 4E binding protein 1; mTOR, mammalian target of rapamycin; p70S6K, serine/threonine kinase; Akt, Protein kinase B; FoxO, Forkhead box O transcription factors; IL-6, interleukin-6; LIF, leukemia inhibitory factor, TNF, tumor necrosis factor, IL-1, interleukin-1, TRAF, TNF receptor associated factor; NF-κB, factor nuclear kappa B; IKK, inhibitor of nuclear factor kappa-B kinase; MAPK, mitogen-activated protein kinase; JAK, janus kinase; STAT, Signal Transducer and Activator of Transcription; ERK, extracellular signal regulated kinase; ALK, activin receptor-like kinase, ACVR2B, activin receptor, SMAD, transcription factor; Atrogin, E3 ubiquitin ligases Muscle Atrophy Fbox (MAFbx); MURF1, Muscle Ring Finger 1, MHC, myosin heavy chain; MCK, muscle creatine kinase; SOCS, Suppressor of cytokine signaling; BCL2/adenovirus E1B 19 kDa protein-interacting protein 3

 Finally Fig. 4 depicts the interactions and cross signaling events among various signaling pathways, for example, myostatin and FOXOs, NF-κB and MURF1, and mTOR that are involved in myotube degradation and myofiber repair via myogenic satellite cells. The precise intersection among these pathways needs to be identified in order to determine key factors controlling atrophy-hypertrophy swifts. Deep dissecting of the essential and separable signaling pathways that regulate these molecular and cellular events are major avenues of future research and promise to improve multiple therapeutic approaches for treating skeletal muscle wasting and cancer cachexia.

## Muscle wasting and cancer cachexia therapy

Various strategies have been studied in order to counteract or prevent catabolic dysfunctions in skeletal muscle in preclinical and clinical trials (Argiles et al. 2014a). Myostatin is well known to act as an inhibitor of muscle mass growth (Glass 2010a, b). The pharmacological blockade of myostatin/activin pathway via the administration of an anti-myostatin monoclonal antibody that inhibit the binding of myostatin to its receptor ActRIIB/ACVR2B was found to be the most effective treatment for muscle wasting in cancer cachetic patients (Lach-Trifilieff et al. 2014). Nonetheless, other studies have shown beneficial effects of the blockage of IL-6 and its signaling pathway in the control of skeletal muscle wasting and cachexia progression in several mouse cancer models (Holmer et al. 2014; Narsale and Carson 2014; Pelosi et al. 2014). Fujita et al. (1996) was the first one to demonstrate that anti-IL-6 receptor antibody prevented skeletal muscle atrophy in colon-26 adenocarcinoma-bearing mice via modulation of lysosomal and ATP-ubiquitin-dependent proteolytic pathways. Next, Zaki and colleagues (Zaki et al. 2004) showed that CNTO 328 (Siltuximab), a monoclonal antibody against IL-6, inhibited human tumor-induced cachexia in Nude mice. The role of IL-6 in the onset of adipose and skeletal muscle wasting has been demonstrated in the in Apc^Min/+^ mouse model. This mouse strain contains a lethal mutation of *adenomatous polyposis coligene* that causes intestinal adenomas (Baltgalvis et al. 2008). Of particular interest, it was shown that the growth of intestinal polyps and intestinal adenomas is associated with the increase in IL-6 circulating levels. Confirming these observations, Tocilizumab, a humanized anti-IL-6 receptor antibody, dramatically attenuated cachexia induced by IL-6 over-expressing lung cancer in animal model. More important, this data was also confirmed in a cohort of patients (Ando et al. 2014). The authors showed that the treatment reduced acute phase protein synthesis in liver while preserved the protein reserves in skeletal muscle of patients (Ando et al. 2014).

**Fig. 5:**
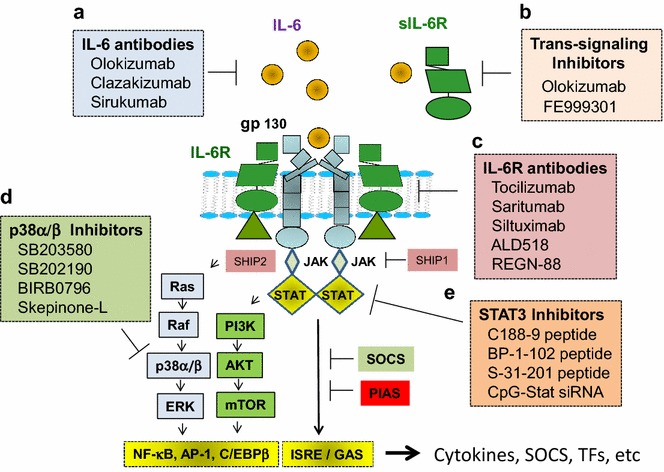
Pharmacological approaches in pre-clinical and clinical trials for treating inflammatory diseases and muscle wasting in cancer cachexia. **a** Chemical and biological inhibitors of IL-6, **b** sIL-6R, **c** IL-6R, and **d** small molecule inhibitors of protein kinases, in the IL-6R downstream signaling pathways. The binding of IL-6 to IL-6R is inhibited by monoclonal antibodies sirukumab, clazakizumab and olokizumab. The interaction of IL-6R with gp130 is blocked by the monoclonal antibodies tocilizumab, sarilumab, ALD518 and siltuximab. FE999301 is a Fc-linked sgp130 recombinant protein that block the interaction of the complex formed by sgp130, soluble IL-6 and IL-6R that act as antagonist of interleukin-6 receptor trans-signaling responses. In box **d** are examples of small molecule inhibitors of p38α/β protein kinase activity with most highly selectivity and in box** e** are examples of phosphopeptide-based prodrugs targeting the SH2 domain of STAT3. SHIP1, SOCS and PIAS are natural negative regulators of JAK/STAT signaling pathways. Abbreviations: PIAS, the protein inhibitors of activated STATs, SOCS, Suppressor of Cytokine Signaling, NF-κB, factor nuclear kappa B, C/EBPβ, Enhancer Binding Protein Beta, AP-1, Activator Protein-1, ISRE, the IFN-stimulatory element, GAS, the IFN-γ-activation site, and TFs, transcription factors

Various humanized monoclonal antibodies to IL-6 and IL-6R have been characterized and are in pre- and clinical trials for many diseases (Fig. 5). These antibodies act at different structural sites of their targets and cause differences side effects when injected in animal models and humans (Shaw et al. 2014; Hunter and Jones 2015). IL-6R trans-signaling pathway is the critical player of IL-6-mediated pathology in autoimmune conditions such as rheumatoid arthritis, colitis, tissue fibrosis and cancer (Wolf et al. 2014). Several clinical studies have proven that treatment with monoclonal antibodies to IL-6R improves symptoms of rheumatoid arthritis, Castleman’s disease and systemic juvenile idiopathic arthritis (Hunter and Jones 2015). The treatment causes side effects such as neutropenia, bacterial super-infection and disruption of gut mucosal integrity due to inhibition of essential innate and adaptive biological activities of IL-6 (Pal et al. 2014; Hunter and Jones 2015).

Various recent studies have explored the possibility of inhibiting muscle wasting and catabolism interfering in the activation of intracellular signaling pathways using small molecules. The STAT3 activation is critically necessary for muscle wasting at downstream of IL-6-induced cachexia in mice bearing C26 colon carcinoma, Lewis lung carcinoma, Apc^Min/+^ colon cancer and B16 melanoma (Bonetto et al. 2012). In fact, blocking STAT3 activation using a cell-permeable STAT3 SH2 domain mimetic peptide (SIP) reduced muscle wasting in mice undergoing cancer cachexia and sepsis (Bonetto et al. 2012; Silva et al. 2015). On the other hand, it is well know that the activation of the JAK/STAT3 downstream pathway is necessary for satellite cell-mediated hypertrophy following acute muscle damage (Toth et al. 2011; Tierney et al. 2014). Thus, the effects of STAT3 inhibitors may be explained on the basis of species differences in STAT3 activation between stem cells from mice and human origin (Hirano et al. 2000; Cornelison 2008; Toth et al. 2011; Do et al. 2014). LIF and IL-6 are required as stem cell growth factors for embryonic stem cell lines derived from mouse strains (Hirano et al. 2000). On the other hand, the human embryonic stem cell lines are only stimulated by the FGF family members, in which FGF2 is the most potent (Do et al. 2014).

Intracellular signals activated by FGFR1 include the MAPK/ERK pathway, also known as the Ras–Raf–MEK–ERK pathway (Cuenda and Rousseau 2007). There are four well-characterized subfamilies of mitogen-activated protein kinases (MAPKs): ERK1/2, ERK5, JNKs and p38s. The canonical activation of p38 MAPKs occurs via dual phosphorylation of the p38MAPK family members: p38α and p38β or p38γ and p38δ (Cuenda and Rousseau 2007). More important, MKK3 and MKK6 (SKK3) are highly selective activator for p38 MAPKs and do not activate JNKs or ERK1/2 (Cuenda and Rousseau 2007). These proteins differ in their expression patterns, substrate specificities and sensitivities to chemical inhibitors. Evidences from a number of studies have established a key role for the p38 MAPK pathway in the conversion of myoblasts to differentiated myotubes during myogenic progression. The activation of p38 mitogen-activated protein kinases p38α and p38β is required for satellite cell proliferation and asymmetric division, which produce one stem and one committed progenitor (Bernet et al. 2014). However, over activation of p38α and p38β due to cellular stress, inflammatory responses and ageing may result in muscle atrophy due to satellite cell depletion or senescence (Brooks et al. 2010; Brien et al. 2013; Tierney et al. 2014). One critical observation in the studies of pharmacologic inhibition of p38α/β using small molecules is that satellite cells can either maintain pluripotency and self-renewal or entering to quiescent state depending on the dose and period of incubation (Brooks et al. 2010; Brien et al. 2013; Tierney et al. 2014). Furthermore, only recently very high selectivity protein kinase inhibitors to p38α and p38β were developed (Koeberle et al. 2012). This may also account for divergences observed in the experiments undertaken with humans and rodent models. From these studies, one can conclude that a major mechanism for muscle wasting is, on the one side, the hyperactivity of IL-6/JAK/STAT1/STAT3 signaling pathway that blocks the expression of genes, such as MyoD, MEF2 and myogenin, and consequently myoblast differentiation and fusion. And, on the other side, the impairment of FGFR1 signaling and hyperactivity of p38α and p38β MAPK signaling that inhibit the proliferation of satellite cells and consequently skeletal muscle self-renewal.

Skeletal muscle atrophy and regrowth are regulated by a complex and context dependent interactions of members of TGF-β superfamily and their receptors as well as extracellular matrix (ECM) protein inhibitors (Glass 2010a, b; Lee et al. 2010; Bonaldo and Sandri 2013; Blau et al. 2015). Bimagrumab or BYM338, a humanized monoclonal antibody to myostatin can reverse cancer cachexia (Glass 2010a, b; Lach-Trifilieff et al. 2014). This antibody binds to activin type II receptors, ActRIIA and ActRIIB, which are primary receptors for myostatin (GDF8), GDF11 and activins. These receptors phosphorylate and activate the type I receptor ALK4 and ALK7, which trigger signaling transduction via Smad2 and 3 transcription factors (Glass 2010a, b, Lach-Trifilieff et al. 2014). GDF11 and myostatin activate the SMAD2/3, p38 MAPK and ERK signaling pathways via the type IIB activin receptor. The development of small molecule inhibitors to ActRII and ALK serine/threonine kinases would be helpful for understanding their roles in distinct signaling pathways. SB-431542 and SB-505124 are examples of small inhibitors that block ALK4, ALK5 and ALK7 kinase activities in use today (Inman et al. 2002; Dacosta et al. 2004). These inhibitors do not inhibit significantly the ERK, JNK, or p38 MAP kinases (Inman et al. 2002; DaCosta et al. 2004). It is interesting that IL-6 activate only STAT3 in skeletal muscle, while myostatin activate SMAD2/3 and STAT3. Therefore, future studies are needed to explore possible pharmacological manipulation of both pathways in order to ameliorate muscle self-renewal while stopping skeletal muscle atrophy.

## Final remarks

IL-6 is a multifunctional cytokine released during inflammatory processes contribute not only to innate and adaptive immune response but also to the complex activation of metabolic and catabolic pathways leading to the increased liver mass and decreased skeletal muscle mass. The local production of IL-6 by skeletal muscle cells and stromal cells promotes activation of satellite cells thereby increasing myotube regeneration. The precise signals by which IL-6 orchestrates intracellular biochemical signaling for skeletal muscle precursor cell proliferation and myotube formation remains controversial. It is possible that unwanted biological effects of IL-6, as that occurring in aging and cancer cachexia, are due to its release chronically and at sustainable levels. The release at low concentration into satellite cell niches IL-6 would promotes repair and regenerates skeletal muscle tissue.

IL-6 binds IL-6Rα forming hetero-complexes with the gp130, which serve as a central signaling module for activation of the canonical JAK/STAT pathway. We know that there is an intense interaction and exchanges of these complexes with several different cytokines in certain pathological conditions. For example, IL-27, LIF and CNTF interaction with both their receptors and gp-130 can transiently inhibit the signaling capacity of other family members, in particular IL-6 (Zvonic et al. 2005; Skiniotis et al. 2008). Alternatively, the undesired effects may be explained by the degradation of the LIFR and protein gp-130 mediated by caspases and lysosomal enzymes (Zvonic et al. 2005; Graf et al. 2008). Finally, we do not know if IL-6 trans-signaling through the soluble IL-6R amplify IL-6 signaling in skeletal muscle of the cachectic patient. The future research to explore the both IL-6 classic signaling and trans-signaling will open new frontiers for design new pharmacological strategies to reverse the atrophy and degeneration of skeletal muscle tissue.

A myriad of factors controls intrinsic and extrinsic signaling pathways leading to satellite cell activation, and proliferation and differentiation. As highlighted in this review, the canonical IL-6/JAK/STAT and FGF/p38 MAPK signaling pathways have both protective and detrimental effects in the skeletal muscle renewal. The deregulation of these signal transduction cascades in inflamed and atrophic muscles may be rescued by single specific kinase inhibitor or combination specific inhibitors for different kinases (Toth et al. 2011; Tierney et al. 2014). Therefore, further research is needed for tailoring or optimizing therapeutic approaches that include monoclonal antibody to cytokines, growth factor and their receptors and small molecules inhibitors of protein kinase signaling pathways to treat muscle wasting and cancer cachexia.
